# Comparative Study of Stress–Strain Behavior and Microstructure of Three Solid-Waste-Powder-Modified Lateritic Clays

**DOI:** 10.3390/ma18102377

**Published:** 2025-05-20

**Authors:** Wei Qiao, Kuncheng Dai, Daming Lin, Bing Yue, Bidi Su, Zhiping Lin, Mingyou Chen, Haofeng Zheng, Zhihua Luo

**Affiliations:** 1 School of Engineering and Technology, China University of Geosciences (Beijing), Beijing 100083, China; 3102230001@email.cugb.edu.cn (W.Q.); 1002202112@email.cugb.edu.cn (K.D.);; 2 Research Institute of Highway Ministry of Transport, Beijing 100088, China; 3 Ningde Sanduao Expressway Co., Ltd., Ningde 352000, China; 4 Fujian Provincial Expressway Group Co., Ltd., Fuzhou 350000, China; 5 Ningde Ninggu Expressway Co., Ltd., Ningde 352000, China; 6 Fuzhou Airport Duplicate Highway Co., Ltd., Fuzhou 350000, China

**Keywords:** lateritic clay, steel slag, ground-granulated blast-furnace slag, fly ash, stress–strain behavior

## Abstract

Lateritic clay is widely distributed in southern China, and its strength is greatly affected by water content. The elevated moisture content in lateritic clay during monsoon periods frequently results in insufficient shear strength for standard engineering applications. Large quantities of solid waste, including steel slag, fly ash, and granulated blast furnace slag, are produced as industrial by-products. This paper is based on the backfilling resource utilization of steel slag, fly ash, and ground-granulated blast-furnace slag as lateritic clay improvement admixtures, along with the stress–strain behavior, strength characteristics, and microstructure of steel-slag-modified lateritic clay, fly-ash-modified lateritic clay, and ground-granulated blast-furnace slag-modified lateritic clay, by combining uniaxial compression tests, straight shear tests, and scanning electron microscopy observation. The experimental results were analyzed to determine the appropriate dosages of three kinds of solid waste and their mechanisms in lateritic clay modification. The results indicate that the unconfined compressive strength of SS-modified lateritic clay exhibited an increase with an increase in SS dosage in the range of 1–7%, the unconfined compressive strength of FA-modified lateritic clay showed an increase with an increase in FA dosage in the range of 1–5%, and the unconfined compressive strength of GGBFS-modified lateritic clay increased with an increase in the use of GGBFS in the range of 1–5%. Under the condition of a 7-day curing age, the unconfined compressive strength of lateritic clay modified with 7% SS increased by approximately 397%, while that modified with 5% FA and 5% GGBFS exhibited increases of about 187% and 185%, respectively. The stress–strain relationship of fly-ash and blast-furnace slag-modified lateritic clays showed elastic–plastic deformation. But the stress–strain behavior of steel-slag-modified lateritic clay at a steel slag dose greater than 5% and a maintenance age greater than 7 days showed elastic deformation. Analyzing the SEM images shows that the more hydration products are generated, the relatively higher the unconfined compressive strength of modified lateritic clay is, and the form of deformation of modified lateritic clay is closer to elastic deformation. Through comparative analysis of modified lateritic clay samples, this study elucidates the property-altering mechanisms of waste powder additives, guiding their engineering utilization.

## 1. Introduction

Lateritic clay, which forms through the laterization of carbonate rocks under specific weather conditions, is widely distributed in tropical and subtropical areas [1,2,3]. Road engineering projects in subtropical and tropical zones of southern China frequently employ lateritic clay as subgrade filling material [4]. The water-holding capacity of lateritic clay originates from its clay-rich composition, resulting in water saturation of particle interfaces [5]. This hydro-mechanical response manifests as pore structure dilation, which compromises the cohesive forces between clay particles and precipitates a marked decline in material strength with rising water content [6,7]. In addition, lateritic clay will have higher compressibility at higher water contents [8,9,10]. The aforementioned characteristics result in problematic differential settlements in lateritic clay roadbeds during rainy periods when subjected to loading. As a remediation strategy, material modification using additives has shown promise. Cement addition, in particular, has been proven to enhance interparticle connectivity and dramatically boost the clay’s cohesive strength [11]. While the cement–lateritic clay mixture offers a practical subgrade option that optimizes local soil use and fulfills engineering needs, cement production remains a carbon-intensive process, generating roughly 7% of global anthropogenic CO_2_ emissions [12]. Nearly 60% of the world’s cement is manufactured in China [13]. If industrial by-products can be utilized for soil modification, the amount of cement required as a soil stabilization additive could be reduced to some extent, thereby indirectly lowering carbon emissions associated with cement production.

Modern industrial processes generate diverse solid waste materials that frequently end up in landfills rather than being recycled. The steel industry, for instance, produces steel slag (SS) and ground-granulated blast-furnace slag (GGBFS) as major byproducts at a rate of 150–200 kg per ton of steel manufactured. Current disposal methods for these materials predominantly involve open storage, creating pressing land-use challenges [14]. While GGBFS demonstrates better recycling performance than SS in China’s metallurgical sector, its annual output of roughly 150 million metric tons still has room for growth [15]. The power sector generates in excess of 160 million tons of FA each year as a coal-fired byproduct. With nearly half going unrecycled, this results in considerable land occupation and contributes to airborne pollution problems [16]. Contrary to being waste materials, these industrial residues demonstrate promising potential for soil reinforcement applications [17,18,19]. Previous studies on lateritic clay modification have demonstrated the effectiveness of incorporating recycled tire rubber particles and GGBFS as soil amendments [20,21]. Replacing cement with industrial byproducts for soil stabilization not only significantly reduces carbon emissions and energy consumption but also enhances waste valorization, thereby alleviating landfill demands and conserving land resources [22].

Studies have been conducted to discuss the mechanisms of SS, FA, and GGBFS in modifying soils. SS—modified dispersive soil with a moisture content of 17% showed enhanced strength due to the formation of calcium silicate hydrate (C-S-H) gels [23]. Fly ash enhances soil stabilization through three primary mechanisms: modifying the soil–water interaction characteristics, restructuring the pore-size distribution, diminishing consolidation-induced deformation, and augmenting shear resistance via cohesive strengthening and controlled strain-softening characteristics [24]. Thus, as the water content of the soil increases, the optimum amount of FA to be used also increases. At a moisture content of 10–15%, GGBFS generated a dense microstructure via Al-Si polymerization, reducing clay plasticity [25].

In this investigation, various types of industrial waste-derived powders were added as additives to lateritic clay and maintained for a period of time, and uniaxial compression tests were conducted on modified lateritic clay to compare the effects of different types of solid wastes, different solid waste mixing amounts, and different maintenance ages on the stress–strain relationship of modified lateritic clay. Three industrial byproduct powders (SS, FA, and GGBFS) were incorporated into lateritic clay as modifiers. The mechanical responses of these amended specimens were benchmarked against untreated lateritic clay to quantify the waste materials’ influence on stress–strain behavior. Meanwhile, the research findings were compared with existing studies on SS-, FA-, and GGBFS-modified soils [26,27,28], and the differences in the ameliorating influences of SS, FA, and GGBFS as additives on lateritic clay and other types of soils were analyzed. In addition, the microstructures of SS-, FA-, and GGBFS-modified lateritic clay were analyzed using a scanning electron microscope to explain the modification mechanism of the three kinds of solid waste powder. This study compared and analyzed the effects of separately incorporating powdered forms of three industrial waste materials (SS, FA, and GGBFS) into lateritic clay on the mechanical properties of the resulting soil mixtures. The findings provide valuable insights into addressing challenges associated with lateritic clay modification in engineering practice.

## 2. Materials and Methods

### 2.1. Materials

#### 2.1.1. Lateritic Clay

**Figure 1:**
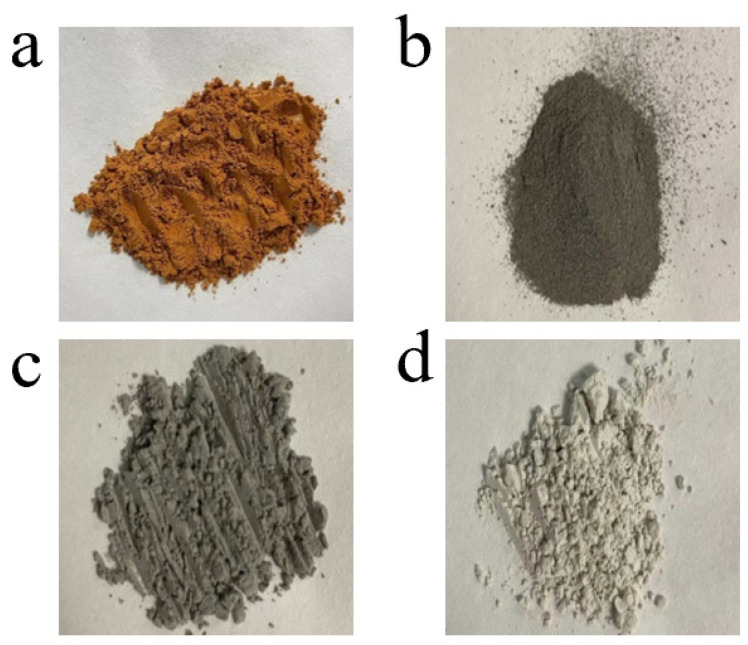
The lateritic clay and solid wastes used in this study: (**a**) lateritic clay; (**b**) SS; (**c**) FA; (**d**) GGBFS.

The lateritic clay used in this work was collected from a newly excavated foundation groove in Jiangxi Province, China. The local climate of the sampling site is a subtropical monsoon climate [29]. Per the Unified Soil Classification System (USCS) [30], the classification of lateritic clay is sandy lean clay (CL). And the natural density of the lateritic clay is obtained as 16.3 g/cm^3^. In order to examine the variation in water content of the lateritic clay in different seasons, samples were taken and water content was measured in the dry seasons (November) and rainy seasons (June), and it was measured that the water content of the lateritic clay was about 12% in the dry season and about 23% in the rainy season. The lateritic clay obtained from the sampling was dried, and then a grinder was used to grind up the dry lateritic clay. The dried and ground lateritic clay was passed through a standard sieve of 0.63 mm aperture, and the sieved lateritic clay was used for the study, as shown in Figure 1a. The Atterberg limits of the lateritic clay were tested according to ASTM D4318 [31], and the results show that the liquid limit of the lateritic clay is 47.7%, the plastic limit of the lateritic clay is 18.1%, and the plasticity index of the lateritic clay is 29.6%.

#### 2.1.2. Solid Waste Powder

**Figure 2:**
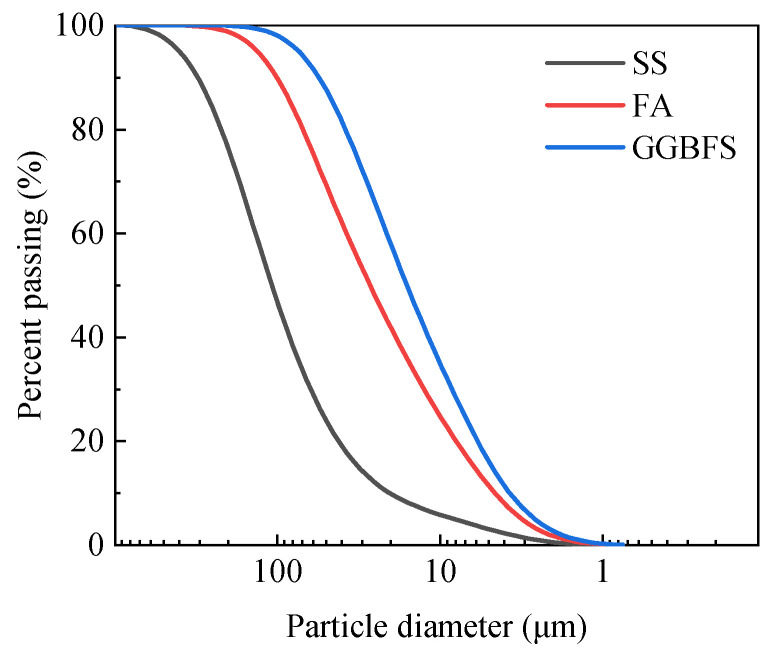
Particle-size distribution of the three solid waste materials.

The SS is chosen from a steel plant in Hebei Province, China. The FA is produced by a thermal power plant in Henan Province, China, and the fuel is brown coal. The GGBFS is collected from a blast furnace slag powder processing plant in Henan Province, China. The collected FA itself is in powder form, and the SS and GGBFS are also ground to powder form, with a fineness of 200 mesh. Figure 1b–d show the three kinds of solid waste powder used in this study. Figure 2 presents the particle size distributions of the three solid waste materials (SS, FA, and GGBFS).

A pH meter was used to test the pH of the three kinds of solid waste powder pulp. Specifically, 10 g of dry sample was weighed and placed in a beaker, and 25 mL of water was added. The mixed pulp was stirred for more than 5 min, after which it was left to stand for 1 h, and the pH value of the pulp was determined. The method for determining the pH of solid waste pulp mentioned later is the same. The pH test was performed on the three kinds of solid waste powder pulp, and the results are shown in Table 1.

**Figure 3:**
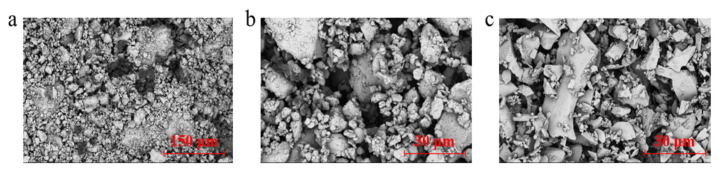
SEM images of the three kinds of solid waste powders: (**a**) SS; (**b**) FA; (**c**) GGBFS.

The scanning electron microscope (Phenom ProX G6, Thermo Fisher, Waltham, MA, USA) was used to observe the micromorphology of the three kinds of solid waste powder. The SEM images of the three kinds of solid waste powders are shown in Figure 3.

In order to examine the relative chemical composition of the three kinds of solid waste, the X-ray fluorescence spectrofluorometer (Series 2, Bruker, Billerica, MA, USA) was used to conduct the X-ray fluorescence (XRF) test on the three solid wastes, and the relative content of potential active ingredients is shown in Table 2. It can be seen that SS has a relative abundance of calcium, FA has a relative abundance of silicon and aluminum, and GGBFS has a relatively high content of calcium, silicon, and aluminum.

**Figure 4:**
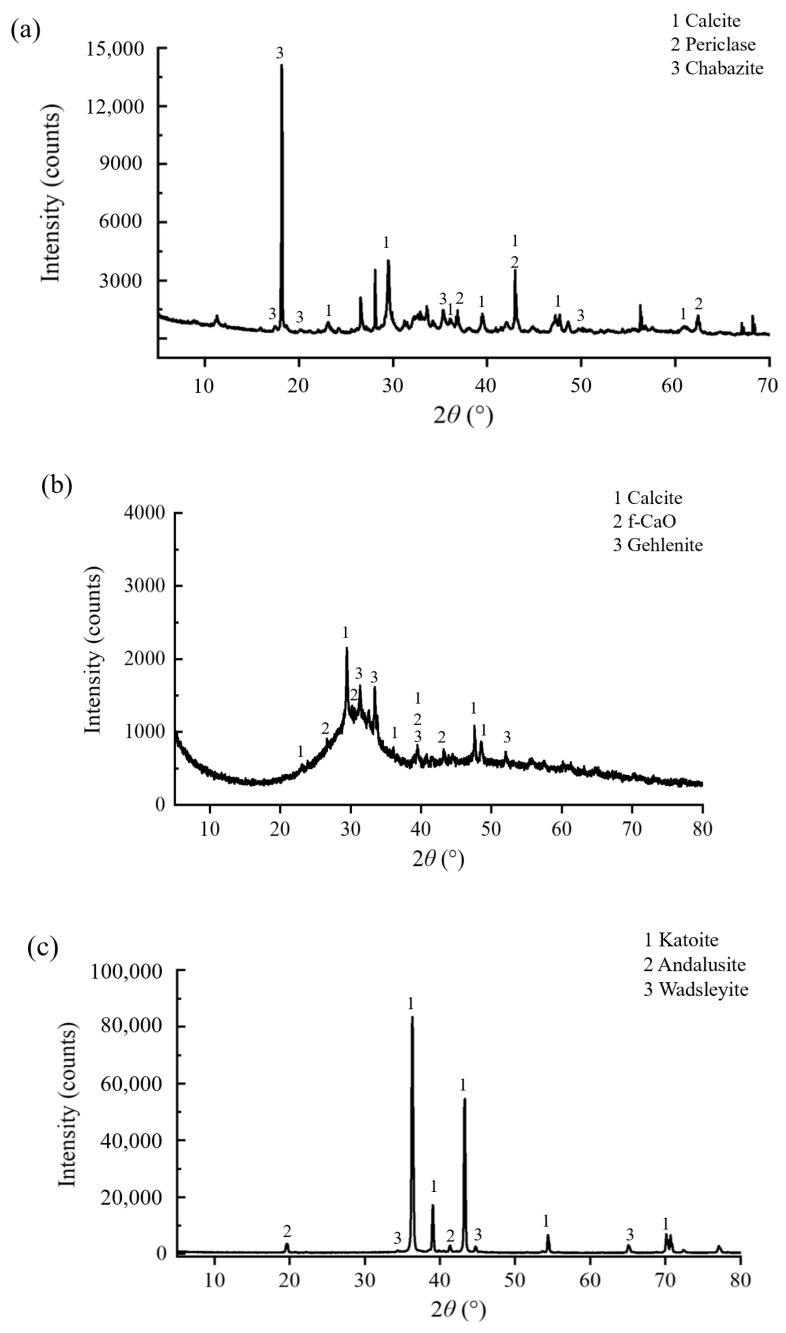
XRD patterns of the three kinds of solid waste: (**a**) SS; (**b**) GGBFS; (**c**) FA.

An X-ray diffractometer (D8 Advance, Bruker) was used to test the mineral composition of Lateritic clay; the diffraction pattern of the three kinds of solid waste is shown in Figure 4. It can be seen that the crystallinity of GGBFS is lower compared to the other two solid waste materials, which is consistent with the SEM images: most of the particles in GGBFS are presented in a glassy state, and the surface of the particles is smoother and denser compared to SS and FA.

### 2.2. Methods

#### 2.2.1. Specimen Preparation

**Figure 5:**
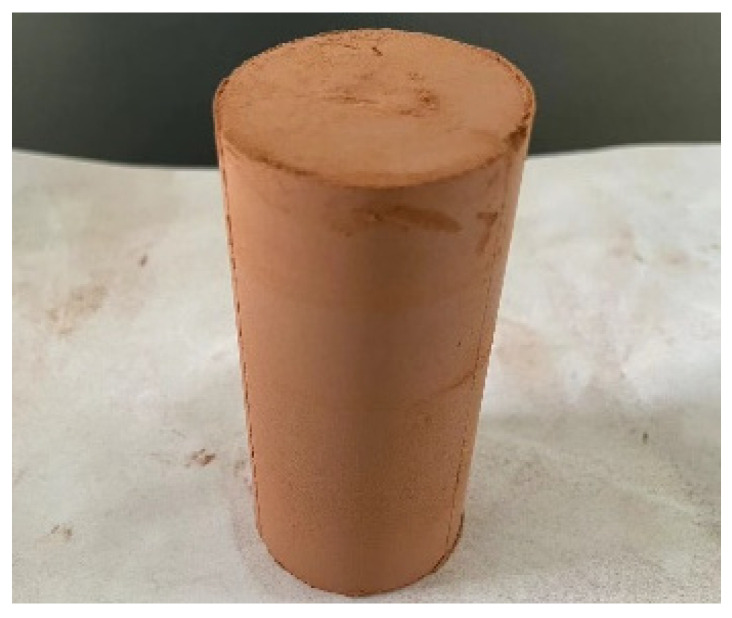
Specimen for uniaxial compression.

All test specimens, including control (unmodified) and treated lateritic clay samples, were fabricated as standard cylinders in compliance with ASTM D4832 requirements [32], with dimensions of 39 mm in diameter and 80 mm in height. The specimen was fabricated using a stainless-steel three-valve mold, and the mold and the finished specimen are shown in Figure 5. The finished cylindrical specimens will be used for uniaxial compression testing.

In making the unmodified lateritic clay specimens, the specimens were made at 5%, 10%, 15%, 20%, 25%, and 30% moisture content, and the dry density of the lateritic clay in each specimen was consistent with the natural density.

When making modified lateritic clay for uniaxial compression tests, the moisture content of the soil should not be too high in order to make the specimens easy to compact and mold. Three technical considerations guided the 15% moisture content selection: (1) Climatic relevance: Approximating but slightly exceeding the material’s typical in situ moisture levels; (2) Practical utility: Ensuring direct transferability to regional construction practices; (3) Manufacturability: Positioned below the plastic limit (PL = 18%) and optimum moisture content (OMC = 17%) to facilitate compaction while preventing excessive plasticity.

After the modified lateritic clay specimens were made and cured, a burette was used to simulate the process of changing the water content of the lateritic clay by rainfall, with a drop of 10% of the mass of the specimen being added to each specimen. Uniaxial compression tests will be conducted on the modified lateritic clay specimens after the addition of water.

After mixing the lateritic clay and solid waste powder in the proportions shown in Table 3, the mixed soil mixture was made into specimens using molds, and the compaction of each specimen was controlled at 93%. Each group of specimens was subjected to three curing ages of 3, 7, and 14 days.

Following preparation, all specimens were cured under controlled conditions (20 ± 2 °C, ≥95% RH) in a standard humidity chamber until reaching the specified curing age.

**Figure 6:**
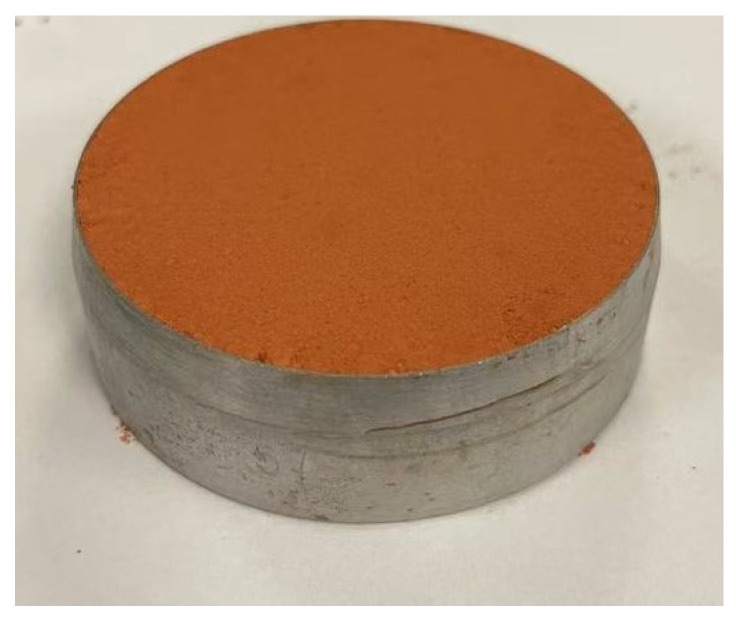
Specimen for direct shear test.

The specimen used for the Direct shear test is a flat, cylindrical specimen with a diameter of 61.8 mm and a height of 20 mm (as shown in Figure 6). During fabrication, the soil sample in the mold is pressed into shape using a hydraulic jack with a compaction of not less than 93%.

#### 2.2.2. Uniaxial Compression Test

After maintenance is completed, the uniaxial compression tests of the modified lateritic clay specimens were conducted on a uniaxial compression tester (Y-300D, Hengle Test Instrument, Jinan, China). The tests were performed at a constant rate of 0.8 mm/s, and the stress–strain curves of the tests were recorded

#### 2.2.3. Direct Shear Test

Direct shear tests were carried out on unmodified lateritic clay with different moisture contents and on lateritic clay modified by different kinds of solid waste powder using a strain control direct shear tester (ZJ quadruple strain control direct shear tester, NJ Soil Instrument, Nanjing, China). Four identical specimens were sheared at normal stresses of 100 kPa, 200 kPa, 300 kPa, and 400 kPa, respectively. The shear rate of the direct shear tester was 0.8 mm/min. The test was considered complete when the shear displacement reached 1/5 of the specimen diameter.

## 3. Results and Discussion

### 3.1. Stress–Strain Behavior

#### 3.1.1. Stress–Strain Behavior of Lateritic Clay with Different Water Contents

**Figure 7:**
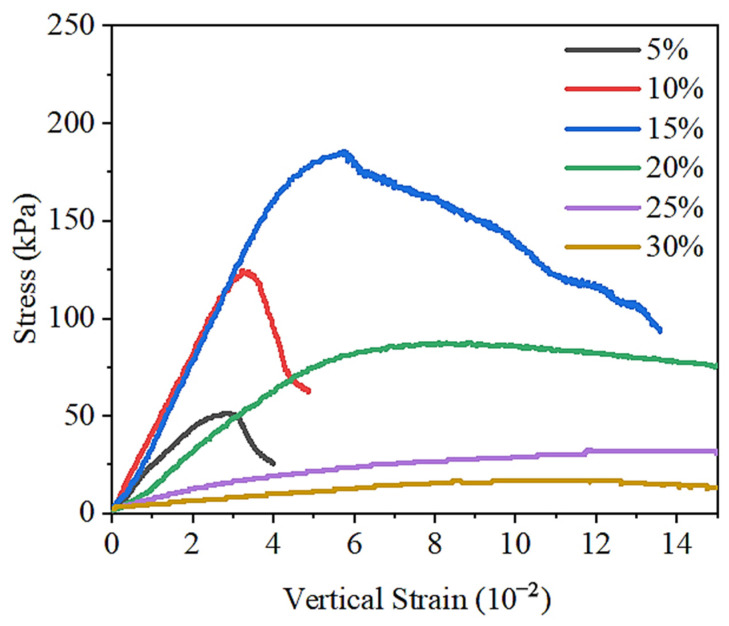
Stress–strain relationships of lateritic clay specimens with different water contents.

The stress–strain relationships for lateritic clay specimens with different water contents under uniaxial compression conditions are given in Figure 7.

For the lateritic clay specimens with 5% and 10% water content, both, in the vertical strain, reached about 3%, the stress reached the maximum value, the peak stress of the lateritic clay specimen with 5% water content was 52 kPa, and the peak stress of the lateritic clay specimen with 10% water content was 123 kPa. Then, the axial stress decreases rapidly after the peak stress is reached, and the form of damage of the specimens is brittle damage.

For the lateritic clay specimen with 15% moisture content, the modulus of elasticity before damage was similar to that of the 10% moisture content specimen, but the strain at which the peak stress was reached was higher, exceeding 5%, while the peak stress also exceeded 180 kPa. The damage form of the specimen with 15% moisture content exhibited brittle damage, and there was a rapid decrease in axial stress after damage, but the decrease was less than that of the red clay specimens with 5% and 10% moisture content.

After the water content of the lateritic clay specimens exceeded the plastic limit of the lateritic clay, the peak stress of the lateritic clay specimens showed a significant decrease. The peak stress of the lateritic clay specimen with 20% moisture content was 88 kPa, the peak stress of the lateritic clay specimen with 25% moisture content was 32 kPa, and the peak stress of the lateritic clay specimen with 30% moisture content was 16 kPa. The 20% moisture content lateritic clay specimens began to gradually decrease in stress after the vertical strain reached 8%, and the 25% and 30% moisture content lateritic clay specimens did not show a significant decrease in stress as the strain continued to increase after the maximum stress was reached. In terms of damage form, the lateritic clay specimens with 20%, 25%, and 30% water content all showed plastic damage.

#### 3.1.2. Stress–Strain Behavior of Lateritic Clay Modified by Different Kinds of Solid Waste

**Figure 8:**
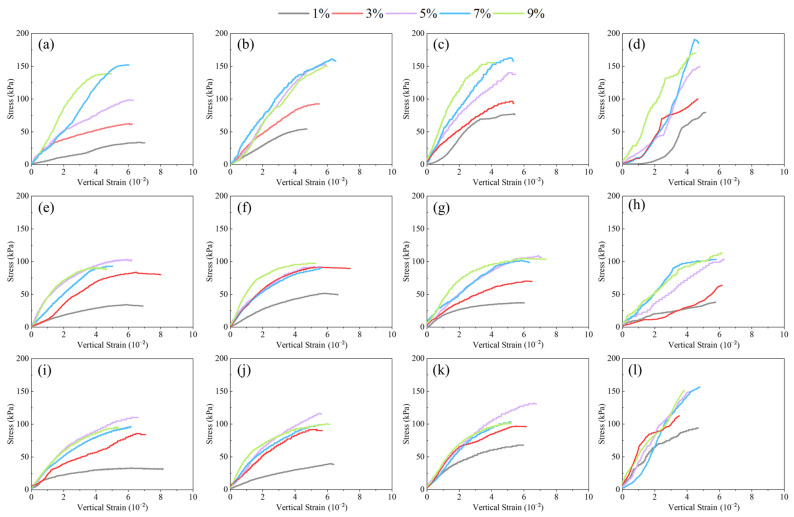
Stress–strain curves of different kinds of solid-waste-modified lateritic clay: (**a**) SS-modified soil maintenance for 3 days; (**b**) SS-modified soil maintenance for 7 days; (**c**) SS-modified soil maintenance for 14 days; (**d**) SS-modified soil maintenance for 28 days; (**e**) FA-modified soil maintenance for 3 days; (**f**) FA-modified soil maintenance for 7 days; (**g**) FA-modified soil maintenance for 14 days; (**h**) FA-modified soil maintenance for 28 days; (**i**) GGBFS-modified soil maintenance for 3 days, (**j**) GGBFS-modified soil maintenance for 7 days, (**k**) GGBFS-modified soil maintenance for 14 days, (**l**) GGBFS-modified soil maintenance for 28 days.

The stress–strain relationships of three kinds of solid-waste-modified lateritic clay obtained by uniaxial compression tests are given in Figure 8.

During the uniaxial compression test, the modified lateritic clay incorporating three types of solid waste materials all exhibited characteristics of elastoplastic failure. Specifically, they first underwent elastic deformation, followed by significant plastic deformation. The specimens showed pronounced lateral bulging until ultimate failure occurred. When failure occurred, all three types of solid-waste-modified lateritic clay specimens developed axial cracks on their lateral surfaces. Among them, the cracks in the SS-modified lateritic clay specimens were notably longer and wider than those in the FA- and GGBFS-modified ones.

Figure 8a shows that for the SS-modified lateritic clay with a maintenance time of 3 days, the plastic deformation of the modified soil has a wider range and exhibits more obvious elastic–plastic deformation characteristics for SS dosages of 1% and 3%. For the modified lateritic clay specimens with SS doping of 5%, 7%, and 9%, the stress increases approximately linearly with an increase in strain at the vertical strains of less than 2%, exhibiting elastic deformation, and enters into elastic–plastic deformation stage at strains of more than 2%. Figure 8b,c show that the deformations of the elastic deformation stage of 5%, 7%, and 9% dose SS-modified lateritic clay increased, and the peak stresses also increased when the maintenance time was increased. Meanwhile, the elastic deformation stage of modified lateritic clay with 3% SS dosing appeared.

Figure 8e–g show that for the FA-modified lateritic clay, the peak strength and deformation modulus of the modified red clay basically show an increasing trend when the addition amount was increased from 1% to 5%. However, after the addition amount of FA reaches 5%, and then an increase in the FA dosage, the peak strength and deformation modulus of the modified lateritic clay no longer significantly increase. The modified lateritic clay with different FA dosages basically exhibits elastic–plastic deformation. For modified lateritic clay with the same FA dosage, there was an increase in the peak stress of the modified lateritic clay with the increase in the age of maintenance, but the increase was small.

Figure 8i–k show that GGBFS-modified lateritic clays, with GGBFS dosages of 5%, 7%, and 9%, are very close to each other in terms of peak stress and deformation modulus. Figure 8h,i show that the peak stresses and deformation moduli of modified lateritic clay with different GGBFS dosages increase to some extent with the increase in the age of maintenance, and the increase in deformation modulus of modified lateritic clay with 3% GGBFS dosage is relatively more obvious.

As shown in Figure 8d,l, the laterite modified by SS and GGBFS exhibits a certain increase in compressive strength and deformation modulus after a longer curing period of 28 days under the dosage condition of 3–9%. In contrast, for the FA-modified laterite, there are no significant changes in compressive strength and deformation modulus after 28 days of curing compared with those under shorter curing ages, such as 7 days and 14 days.

Based on the results of the uniaxial compression test, and considering the time cost of maintenance and the effect of modification, the three kinds of solid-waste-modified lateritic clay with a maintenance age of 7 days were chosen to be more representative for the subsequent tests. At a maintenance age of 7 days, for SS-modified lateritic clay, the unconfined compressive strength of modified lateritic clay was relatively the highest at an SS dosage of 7%. In the FA-modified lateritic clay, the highest unconfined compressive strength of modified lateritic clay was observed at 9% FA dosage. Among the lateritic clay modified with GGBFS, the highest unconfined compressive strength of modified lateritic clay was observed at 5% GGBFS dosage.

In comparison with other research findings, the study by Xu et al. [26] demonstrates that incorporating approximately 40% SS significantly enhances the shear strength of clay. This dosage is notably higher than the optimal SS addition identified in this study. The discrepancy may be attributed to the fact that the SS particle size used in previous experiments was considerably larger than the steel slag powder adopted in this research. Particle size influences the utilization efficiency of SS to some extent, and refining SS into finer particles can improve its effectiveness, thereby reducing the required dosage. The experimental results from Zheng et al. [27] indicate that the optimal dosage of FA for modifying gravel layer soil is 15%, which is higher than the optimal FA dosage for red clay modification identified in this study. This difference arises because the porosity of the gravel layer soil is significantly greater than that of red clay, necessitating a higher FA dosage to fulfill its pore-filling role. The findings of Darsi et al. [28] suggest that adding around 10% GGBFS substantially improves the strength of high liquid limit clay. However, the proposed optimal GGBFS dosage for red clay modification in this study is lower. This is because the liquid–plastic limit of the red clay in this research is relatively low. Excessive GGBFS addition would result in insufficient moisture for complete hydration of the slag. Unhydrated GGBFS particles would instead act as barriers between soil particles, negatively impacting the strength of the modified soil.

The experimental results show that the SS-modified lateritic clay with 7% and 9% SS dosages and the GGBFS-modified lateritic clay with 7% and 9% GGBFS dosages, after 28 days of curing, meet the recommended strength values for Controlled Low-Strength Materials (CLSM) specified in ACI 229R-13 (Report on Controlled Low-Strength Materials), indicating their application potential in non-load-bearing backfill projects. Compared with directly using lateritic clay for backfilling in non-load-bearing engineering, using modified lateritic clay offers significant advantages. Existing research results indicate that the soil mixed with solid waste powder represented by SS can maintain higher structural stability when exposed to water compared to unmodified soil [33]. Meanwhile, the soil modified with solid waste powder exhibits a certain level of long-term strength, but this strength does not become excessively high to hinder subsequent excavation [34]. This is because the strength of backfilled natural lateritic clay depends on its compaction degree, and is prone to decrease due to moisture content fluctuations and uneven compaction. Therefore, backfilling with natural soil requires strict control of moisture content, which makes construction difficult in areas with high groundwater levels or during rainy seasons. In this study, both SS-modified lateritic clay and GGBFS-modified lateritic clay can maintain relatively stable strength across a wide range of moisture contents, making them more suitable for diverse construction scenarios. Additionally, the volumetric stability of the SS-modified lateritic clay is significantly improved, which is beneficial for the structural stability of engineering structures (such as pipelines) that are backfilled.

### 3.2. Shear Strength

**Figure 9:**
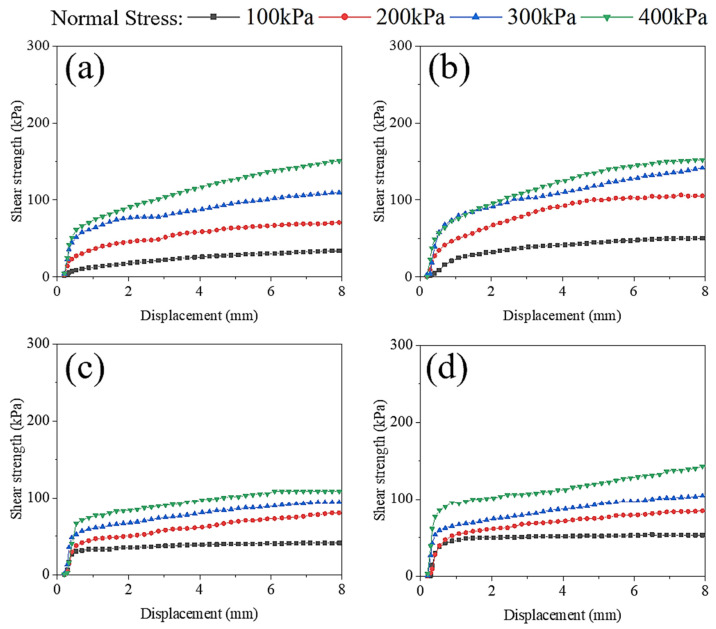
Shear stress–displacement relationship curves for modified and unmodified lateritic clay: (**a**) Unmodified lateritic clay with 25% water content; (**b**) SS-modified lateritic clay; (**c**) FA-modified lateritic clay; (**d**) GGBFS-modified lateritic clay.

SS-modified lateritic clay specimens with 7% SS dosing, FA-modified lateritic clay specimens with 9% FA dosing, and GGBFS-modified lateritic clay specimens with 5% GGBFS dosing were produced for straight shear tests, respectively. Lateritic clay with 25% moisture content was used to make solid-waste-modified lateritic clay specimens. Specimens were subjected to 7-day moisture curing at 20 ± 2 °C and >95% relative humidity in an environmental chamber. After the maintenance was completed, the specimens were taken out for direct shear tests, and as a comparison, the direct shear test was performed on unmodified lateritic clay with 25% water content, and the results of the direct shear test are shown in Figure 9.

**Figure 10:**
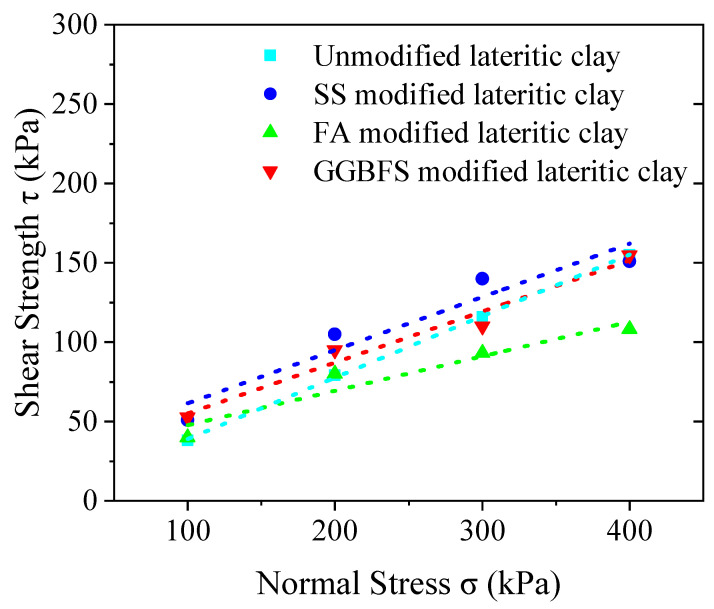
The relationship of shear strength–vertical unit pressure for modified and unmodified lateritic clay.

The peak points or stable values on the shear stress–displacement relationship curves as the shear strength were selected, the shear strength as the vertical axis and the vertical unit pressure as the horizontal axis on the coordinate graph were used, and the four points obtained from testing four parallel specimens in each group of modified or unmodified lateritic clay to obtain a straight line were fitted. The inclination angle of the straight line is the internal friction angle *φ* of the modified or unmodified lateritic clay, and the intercept of the straight line on the vertical axis is the cohesive force *c* of the modified or unmodified lateritic clay. The fitted relationship between shear strength and vertical unit pressure is shown in Figure 10.

The cohesive force and friction angle of 25% water content lateritic clay added with SS, FA, and GGBFS, respectively, and cured for 7 days, are given in Table 4. As a comparison, the cohesive force and friction angle of unmodified 25% water content lateritic clay are given together in Table 4.

Comparing the results of straight shear tests of lateritic clay with different modification conditions, it can be found that the cohesive force of SS-, FA-, and GGBFS-modified lateritic clay after 7 days of maintenance has increased to some extent. Among the three kinds of solid waste material, SS showed the greatest increase in cohesive force. For SS- or GGBFS-modified lateritic clay, the cohesive force is closer to that of unmodified lateritic clay. In contrast, the angle of internal friction of FA-modified lateritic clay decreased considerably compared to that of unmodified lateritic clay.

### 3.3. Relationship Between the Microstructure and Stress–Strain Behavior of Modified Lateritic Clay

**Figure 11:**
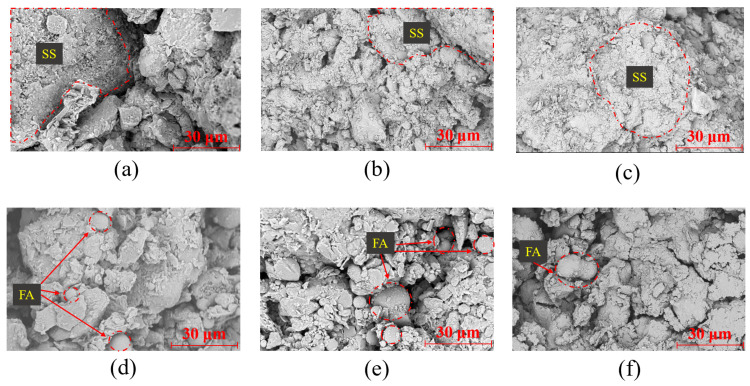

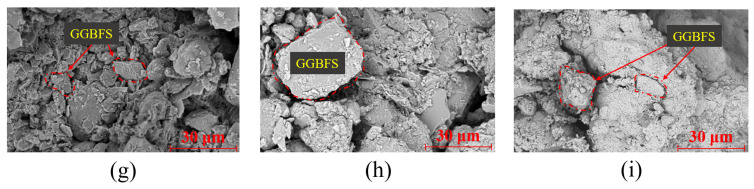
SEM images of different kinds of solid waste modified lateritic clay: (**a**) SS-modified soil maintenance for 3 days; (**b**) SS-modified soil maintenance for 7 days; (**c**) SS-modified soil maintenance for 14 days; (**d**) FA-modified soil maintenance for 3 days; (**e**) FA-modified soil maintenance for 7 days; (**f**) FA-modified soil maintenance for 14 days; (**g**) GGBFS-modified soil maintenance for 3 days; (**h**) GGBFS-modified soil maintenance for 7 days; (**i**) GGBFS-modified soil maintenance for 14 days.

According to the results of uniaxial compression tests, the relationship between solid waste doping and the modification effect in solid-waste-modified lateritic clay of different ages was considered comprehensively, and the SS-modified lateritic clay specimens with 7% SS dosing, the FA-modified lateritic clay specimens with 5% FA dosing, and the GGBFS-modified lateritic clay specimens with 5% GGBFS dosing were produced, respectively. SEM was used to observe the solid-waste-modified lateritic clay specimens made according to the above dosages. The microstructures of SS-, FA-, and GGBFS-modified lateritic clay after 3 days, 7 days, and 14 days of maintenance are given in Figure 11.

Figure 11a–c show the SEM images of modified laterite with 7% SS dosage at different maintenance times. For the SS-modified lateritic clay with a maintenance age of 3 days, the contours of the SS particles and laterite particles can be clearly distinguished in the SEM images. The SS-modified lateritic clay with a maintenance age of 3 days showed bonding of the lateritic clay particles, and there was still a relatively large number of pores in the modified soil. For SS-modified lateritic clay with a maintenance age of 7 days, a certain amount of gelatinous hydration products was produced in the soil, which bonded the particles and filled the pores in the modified lateritic soil. The size of the pores in the SS-modified lateritic clay with a maintenance age of 7 days was significantly reduced compared to the SS-modified lateritic clay with a maintenance age of 3 days. For the SS-modified lateritic clay with a maintenance age of 14 days, the microstructure was denser compared to the SS-modified lateritic clay with maintenance ages of 3 and 7 days, and a large amount of gelatinous hydration products filled most of the pores in the lateritic clay. After curing the SS-modified lateritic clay for a certain period, the hydration products generated in the soil fill most of the pores, and these hydration products produce obvious bonding between soil particles. Therefore, the cohesion of the SS-modified lateritic clay obtained through direct shear tests is significantly improved.

Figure 11d–f show the SEM images of modified lateritic clay with 5% FA dosage at different maintenance times. No significant generation of hydration products was observed in FA-modified lateritic clay at 3-, 7-, and 14-day maintenance ages. For the FA-modified lateritic clay at 7- and 14-day maintenance age, some scaly material can be observed on the surface of the FA particles, which may have been produced by the hydration of the FA itself. Due to the insufficient content of CaO in FA, only a small amount of C-S-H gel could be generated on the surface of FA particles, which was not enough to produce extensive cementation between FA particles and lateritic clay particles. It can be found from the SEM images of the fly ash that the fly ash mainly consists of a large number of regular spherical particles. After the addition of fly ash to the lateritic clay, the pores of the lateritic clay are filled with fly ash microbeads, which act as a certain lubricant and reduce the internal friction angle of the modified clay [35].

Figure 11g–i show the SEM images of modified lateritic clay with 5% GGBFS dosage at different maintenance times. In the GGBFS-modified lateritic clay with a maintenance age of 3 days, it can be observed that the GGBFS particles were filled between the laterite particles, and there was no visible reaction product generation on the surface of the GGBFS particles. The more obvious hydration phenomenon was observed in the GGBFS-modified lateritic clay with a maintenance age of 7 days, and there were flaky hydration products produced on the surface of the GGBFS particles. The hydration of GGBFS particles in the GGBFS-modified lateritic clay at 14 days of maintenance was more pronounced, which produced a certain amount of hydration product gels. The hydration product gels had a filling effect on the pores between the GGBFS and laterite particles. In the SEM images, the hydration products generated around the GGBFS particles can also produce a certain cementing effect on the lateritic clay particles. Therefore, in direct shear tests, the cohesion of GGBFS-modified lateritic clay is significantly higher than that of unmodified lateritic clay. Regarding the filling effect on the pores in lateritic clay, the pore filling rate in GGBFS-modified lateritic clay is higher than that in FA-modified lateritic clay but lower than that in SS-modified lateritic clay. A higher pore filling rate means a larger effective friction area when internal displacement occurs in the modified lateritic clay, resulting in the internal friction angle of GGBFS-modified lateritic clay being between those of SS-modified lateritic clay and FA-modified lateritic clay.

Comparing the SEM images of the three kinds of solid-waste-modified lateritic clay, it can be seen that the amount of hydration products in the GGBFS-modified lateritic clay is more than that of the FA-modified lateritic clay, but less than that of the SS-modified lateritic clay. SEM images show that after the addition of three solid waste materials to lateritic clay, the pore-filling ability in the soil, from high to low, is in the order of SS, GGBFS, and FA. In SS-modified lateritic clay, pores with a diameter of over 20 μm are basically filled with hydration products after 7 days of curing, and pores with a diameter ranging from 5 μm to 20 μm are also almost completely filled after 14 days of curing. In GGBFS-modified lateritic clay, pores with a diameter of over 20 μm are basically filled after 14 days of curing, but there are still many pores with a diameter of less than 20 μm. In FA-modified lateritic clay, even after 14 days of curing, although the number of pores with a diameter of over 20 μm has decreased, they still exist.

The SEM images reveal that in terms of the formation rate of hydration products, the lateritic clay modified by the three solid waste materials exhibits different stages—specific characteristics. In the SS-modified lateritic clay, the formation of hydration products through the hydration reaction can be clearly divided into two stages. For the SS-modified lateritic clay cured for 14 days, the amount of hydration products formed in the first 7 days is significantly higher than that in the last 7 days. In the FA-modified lateritic clay and GGBFS-modified lateritic clay, the increasing rate of the amount of hydration products is more similar to a linear increase.

By analyzing the relationship between the dosage of solid waste materials and the mechanical properties of modified lateritic clay in combination with the microstructure of the modified laterite shown in SEM images, it can be seen that for SS-modified lateritic clay, the improvement in mechanical properties mainly relies on the bonding and filling effects of hydration products generated by steel slag. Therefore, increasing the SS dosage can enhance the production of hydration products, thereby improving the mechanical properties of SS-modified lateritic clay. SEM images show that after adding 7% SS, the amount of generated hydration products is sufficient to fill the soil pores. Therefore, further increasing the SS dosage no longer significantly improves the mechanical properties of SS-modified soil. For FA- and GGBFS-modified lateritic clay, their internal hydration reactions are relatively slow. Under curing ages below 7 days, the improvement in mechanical properties of modified lateritic clay mainly depends on the filling effect of solid waste materials, while the bonding effect brought by hydration products of solid waste materials plays a more auxiliary role. However, there is an upper limit to the amount of solid waste materials that can provide a filling effect in modified lateritic clay, which is related to soil porosity. Exceeding this limit, more solid waste materials cannot further densify the soil. This is why the optimal dosages of FA and GGBFS are around 5%; beyond this dosage, excessive FA and GGBFS can no longer densify the modified lateritic clay and may negatively affect its strength to a certain extent due to their own buoyant bead effects.

### 3.4. Volumetric Stability of Modified Lateritic Clay

**Figure 12:**
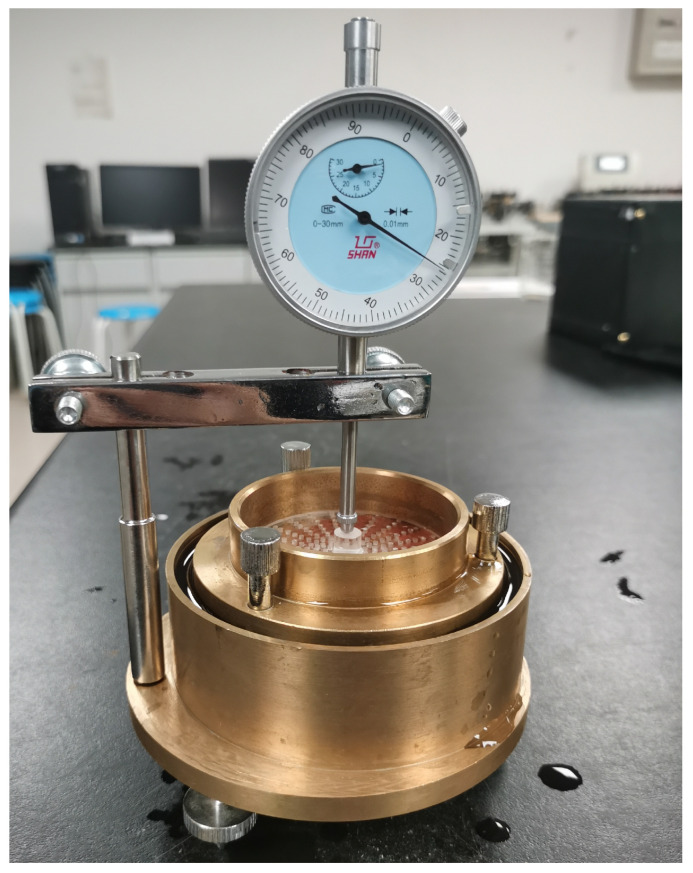
Unloaded expansion meter.

In addition to affecting the strength of lateritic clay, water also impacts its volumetric stability. As mentioned in Section 3.1.2, SS-modified lateritic clay with 7% or 9% SS dosages and GGBFS-modified lateritic clay with 7% or 9% GGBFS dosages can meet the strength requirements for non-load-bearing backfilling engineering. To further investigate the effect of the modification process on the volumetric stability of lateritic clay, unmodified lateritic clay, 7% SS-modified lateritic clay, and 7% GGBFS-modified lateritic clay were made into cylindrical specimens with a diameter of 61.8 mm and a height of 20 mm, as shown in Figure 6. An expansion meter (as shown in Figure 12) was used to measure the vertical expansion rate of the unmodified lateritic clay specimen, the 7% SS-modified lateritic clay specimen after 7 days of curing, and the 7% GGBFS-modified lateritic clay specimen after 7 days of curing.

The results showed that the expansion rate of the unmodified lateritic clay specimen was 25%, that of the SS-modified lateritic clay specimen was 9%, and that of the GGBFS-modified lateritic clay specimen was 36%. This indicates that the addition of SS not only improves the strength of lateritic clay but also significantly enhances its volumetric stability. The ion exchange effect of iron ions in SS densifies soil particles. In contrast, due to its higher content of free calcium oxide, GGBFS tends to react with water to form loose calcium hydroxide, causing expansion. Considering the stability requirements of backfilling engineering, mixing lateritic clay with SS before backfilling is a better choice.

## 4. Conclusions

This study analyzed the stress–strain relationships of lateritic clay modified with different dosages of SS, FA, and GGBFS under different curing conditions. The following conclusions can be obtained by combining the mechanical test results and SEM images of modified lateritic clay.

(1) The unconfined compressive strength of SS-modified lateritic clay exhibited an increase with increasing SS dosage in the dosage range of 1–7%, and the percentage of elastic deformation stage in the deformation of SS-modified lateritic clay under uniaxial compression became larger with increasing maintenance age and SS dosage.

(2) The unconfined compressive strength of FA-modified lateritic clay showed an increase with an increase in FA dosage in the range of 1–5%. The deformation form of FA-modified lateritic clay remained elastic–plastic under the conditions of different FA dosages and maintenance ages.

(3) The unconfined compressive strength of GGBFS-modified lateritic clay increased with the increase in the use of GGBFS in the range of 1–5%. The deformation modulus of GGBFS-modified lateritic clay increased with increasing maintenance age.

(4) After the addition of the three kinds of solid waste to lateritic clay, the cohesion of the modified lateritic clay showed a substantial increase compared to the unmodified lateritic clay, and the angle of internal friction of the FA-modified lateritic clay showed a relatively significant decrease compared to the unmodified lateritic clay.

(5) Comparing the amount of hydration products generated in the three kinds of solid-waste-modified lateritic clay at the same maintenance age, most hydration products were generated in the SS-modified lateritic clay, the least in the FA-modified lateritic clay, and the amount of hydration products generated in the GGBFS-modified lateritic clay was between those of the SS-modified lateritic clay and the FA-modified lateritic clay. SS mainly relies on the bonding of laterite particles by hydration products generated through hydration reactions to improve the mechanical properties of lateritic clay, while the improvement in the mechanical properties of lateritic clay by FA and GGBFS originates from the combined effects of filling and bonding.

## Figures and Tables

**Table 1:** pH of the solid wastes and the Lateritic clay pulp.

Different Kinds of Pulp	pH Value
SS	12.80
FA	8.18
GGBFS	9.50
Lateritic clay	6.69

**Table 2:** Chemical composition of the Lateritic clay and the three kinds of solid wastes.

Oxides	Composition (wt.%)			
	Lateritic Clay	SS	FA	GGBS
Calcium oxide (CaO)	0.74	41.22	3.60	59.31
Silicon dioxide (SiO_2_)	52.99	6.32	35.71	16.31
Aluminum oxide (Al_2_O_3_)	18.78	2.88	37.34	10.24
Ferric oxide (Fe_2_O_3_)	13.48	22.44	9.86	1.46
Magnesium oxide (MgO)	1.04	5.68	0.46	5.63

**Table 3:** Grouping of Specimen Preparation for Modified Lateritic Clay.

Additive	Test Group	Dry Mass Ratio of Solid Waste to Lateritic Clay	Curing Time (Days)
SS	1-1	0.01	3, 7, 14
	1-2	0.03	
	1-3	0.05	
	1-4	0.07	
	1-5	0.09	
FA	2-1	0.01	3, 7, 14
	2-2	0.03	
	2-3	0.05	
	2-4	0.07	
	2-5	0.09	
GGBFS	3-1	0.01	3, 7, 14
	3-2	0.03	
	3-3	0.05	
	3-4	0.07	
	3-5	0.09	

**Table 4:** Chemical composition of the laterite and the three kinds of solid wastes.

Specimen	Cohesive Force (kPa)	Friction Angle (°)
Unmodified lateritic clay	3	21
SS-modified lateritic clay	28	19
FA-modified lateritic clay	26	12
GGBFS-modified lateritic clay	23	18

## Data Availability

The original contributions presented in this study are included in the article. Further inquiries can be directed to the corresponding author.
